# Volunteer Experiences of a School‐Based Volunteer Program

**DOI:** 10.1002/hpja.956

**Published:** 2025-03-11

**Authors:** Sharyn Burns, Hanna Saltis, Jacqueline Hendriks, Jenny Tohotoa, Christina Pollard

**Affiliations:** ^1^ Collaboration for Evidence, Research and Impact in Public Health, School of Population Health, Curtin University Bentley Western Australia Australia

**Keywords:** emotional and academic impacts, school‐based intervention, school‐based mentors, school‐based volunteers, student social, Volunteer, volunteer wellbeing

## Abstract

**Issue Addressed:**

Individuals engage in volunteer activities due to a range of intrinsic, extrinsic, and altruistic factors; and this can have a positive impact on their subjective wellbeing. Within a school context, mentoring programs can connect adult volunteers with students, to provide social and/or academic support; however, evaluation data related to these programs is limited and often focused on student perspectives. This paper explores EdConnect volunteers' perspective on volunteering in primary and secondary schools in Western Australia and Victoria.

**Methods:**

A mixed methods evaluation framework utilised surveys (*n* = 380) and telephone interviews (*n* = 22) with Edconnect Mentor and/or Learning Support volunteers.

**Results:**

Most survey respondents reported that volunteering enhanced their sense of community, mental wellbeing, physical health, had helped them to develop new skills. Recurrent themes related to (a) Structural impacts of motivations; and (b) Motivations and inter‐and intrapersonal impacts of volunteering were identified. Various factors impacted the volunteer experience, including volunteer roles and responsibilities, volunteer qualities, school support and training opportunities. Key motivating factors for volunteering were a desire to give back to society, helping students and staff, and contributing to student social, emotional, and academic outcomes.

**Conclusion:**

Volunteers identified a range of academic, social, emotional, and behavioural gains for the students they supported; as well as range of personal benefits for themselves. Despite the positive impact that a school‐based mentoring program can have for key stakeholders, challenges to program implementation do exist and require careful management.

**So What?:**

School‐based mentoring programs are an efficacious way to strengthen partnerships between schools and their local community, with positive benefits for the school, the students, and the adult volunteers. They also contribute to the notion of a ‘school as a community hub’.

## 1 Introduction

Volunteering is often regarded as the act of helping others. It is work that is unpaid, non‐compulsory, not directly associated with family obligations and frequently occurs within a specific time frame [1, 2]. Individuals volunteer for a range of reasons that may be associated with helping others, giving back to society, and gaining additional skills and experience [1]. Consistently, studies highlight intrinsic, extrinsic and altruistic motivations for volunteering [1, 3, 4, 5]. People who are intrinsically motivated to volunteer do so because the activity provides satisfaction and pleasure and is not dependent on external pressures or incentives [3]. Extrinsically motivated volunteers undertake volunteering due to external incentives and rewards. This may include rewards in terms of career and/or psychosocial development [4]. Altruistically motivated people volunteer to increase the welfare of others [5]. University student volunteers have been found to benefit by developing new skills and enhancing career opportunities [4] while studies of older volunteers suggest volunteering can contribute to increased social participation [6]. For all generations, volunteering offers the opportunity to acquire new skills, interests, networks, a renewed sense of purpose and potential pathways for employment.

Subjective wellbeing which refers to how individuals evaluate their lives [7] has been associated with enhanced life outcomes including health status, social relationships, higher productivity and increased educational achievements [8]. A rapid review of 158 studies between 2008 to 2020 found most formal volunteering conducted through organisations, clubs and groups to be beneficial to subjective wellbeing [9]. This review, along with another review of 152 studies in Australia [1] conferred that while volunteering has been found to be positively associated with wellbeing, people with higher levels of wellbeing were more likely to become volunteers. This may also be associated with social capital whereby those with higher levels of social connection and the ability to collaborate and cooperate with others are more likely to volunteer [1]. Conversely, some volunteering, without adequate support of matching volunteer skills to tasks, was associated with anxiety, stress and burnout [9], highlighting the need for support for volunteers.

School‐based mentoring or volunteer programs involve regular meetings between volunteer mentors and students on school grounds [10]. In mentoring, an adult is paired with a student with the goal of fostering a trusting and supportive relationship. The mentoring relationship affords students the chance to seek guidance, problem‐solve, and build self‐esteem and resiliency [11]. Responding to community needs, school‐based mentor and volunteer programs can fill a gap that parents and schools often cannot, in preparing and equipping young people in difficult circumstances with necessary social, academic and life skills [12].

Published research evaluating school‐based mentoring programs is limited and is predominantly focused on student outcomes. Randolph and Johnson (2008) reviewed eight school‐based mentoring programs and found a lack of data on dose and program outcomes [13]. Some studies, mostly in the United States of America (US), have found some evidence of positive impacts on student academic outcomes and behaviour. For example, the *Youth Friends* program based in Kansas and Missouri in the US found statistically significant improvements among Grade 1 and 2 students for community connectedness, goal setting and academic performance for those with low baseline scores [14]. Another US school‐based mentoring program found significant improvements in teacher‐rated academic achievement and student self‐reported academic efficacy of mentored students [10]. A meta‐analysis of three US mentoring programs reported lower rates of absenteeism and improved peer support but not academic achievement [15]. However, a more recent study cautioned that evaluations of school‐based programs often have small effects [16].

EdConnect Australia is a charity that trains, supports and places volunteers in schools in Western Australia and Victoria with the aim of supporting disadvantaged and at‐risk students and the broader school community [17]. The organisation brings together schools, students and volunteers and aims to positively impact each group. Volunteers work with students who may benefit from social, emotional, and/or educational support. The EdConnect program provides two different volunteering opportunities: as a Mentor, whereby the volunteer works with a specific student regularly; and as a Learning Support Volunteer who works in the classroom with small groups or individuals to support the teacher. To better understand the impact of school‐based volunteers an independent evaluation of the EdConnect program was conducted. This paper explores school‐based mentors and volunteers' perspectives on the impact of the EdConnect volunteer program on themselves as volunteers and on the students, they work with.

## 2 Method

### 2.1 Study Overview

A mixed methods approach was adopted comprising a short survey and interviews with a purposive sample of mentors and learning support volunteers. All data collection occurred between October and December 2022. The first phase of this project focused on the development of survey tools for the broader project. Mentor and Learning Support Volunteer (hereafter volunteers) tools were informed from two focus group discussions held with 18 volunteers.

### 2.2 Survey

Volunteers were invited to complete a short survey to understand their experience as a volunteer during the school year. All registered volunteers were contacted via email and provided an information sheet. Two follow‐up emails were sent. Consenting participants completed the survey online via Qualtrics or paper. The majority were completed online; hard copies were placed in a sealed envelope and collected or mailed from the school. The survey took 10 to 15 min to complete. Survey participants were invited to enter a prize draw (6 × AUD$100 gift vouchers).

Survey questions included in this paper relate to demographics and volunteer perceptions of how the program impacted their own wellbeing and feelings of connectedness. Volunteers were asked four questions related to their overall wellbeing (Likert scale; 5 options; strongly agree to strongly disagree) (Table 2). These questions were revised from a previous iteration of the Mentor survey [18] and were tested for face and content validity during formative research with volunteers (*n* = 18). Open‐ended questions provided opportunity for participants to comment on their experiences as a volunteer.

### 2.3 Interviews

At the conclusion of the survey, participants were offered the opportunity to participate in an in‐depth one‐on‐one interview. This information was removed from the data and stored securely as per the ethics board requirements. Potential interview participants were initially contacted via email and were provided with an information sheet and consent form. All interviews were conducted by telephone by one experienced researcher. Interviews lasted between 10 and 30 min. Participants were offered an AUD$50 voucher to thank them for their time. Interviews were audio recorded, transcribed verbatim.

The semi‐structured interview guide was informed by the literature and formative research with volunteers. To gain an understanding of volunteer experience questions initially explored the role of the volunteer and/or mentor before exploring the impact of the EdConnect program on volunteers and students.

### 2.4 Data Analysis

Univariate analyses were conducted via SPSS V21 to analyse quantitative data. Qualitative data were analysed using reflexive thematic analysis. Using Braun and Clarke's six phases' data familiarisation and initial codes and then themes were generated, followed by developing and reviewing themes; refining, defining and naming themes; and writing up [19].

Confirmability and dependability were achieved by ensuring the data analysis process was conducted with the research team allowing for ongoing discussion confirmation of code and themes [20]. Themes were formulated using an inductive approach; hence data were coded without a pre‐existing coding frame. Initial codes were generated. Line‐by‐line analysis was employed to generate descriptive codes and words and phrases were explored to determine shared meanings and perceptions. Using the initial codes, the authors summarised codes into meaningful themes. Original data were reviewed against preliminary themes before final themes were defined and named [19]. A detailed description of the research methods and results was maintained to ensure transferability [20]. Transcriptions and coding were managed using the NVIVO V.12 software [21]. Data were de‐identified, and participants were coded with a number and by gender, for example, P1F.

## 3 Results

### 3.1 Survey Findings

#### 3.1.1 Demographics

Of the 857 active EdConnect Volunteers in Western Australia (*n* = 648) and Victoria (*n* = 209) sent a request to participate in the 2022 Annual Volunteer Survey, 338 (39%) responded. In Western Australia, 37% of volunteers responded (*n* = 240), while Victoria recorded a 33% response rate (*n* = 68). Overall, the response rates reflect the broader distribution of EdConnect volunteers across the two states.

The sample was mostly female (73.7%), aged over 55 (75.7%), Western Australian (71%), based in metropolitain areas (70.4%), and comprised of slightly more Learning Support Volunteers than any other type of volunteer (41.4%). The majority were experienced volunteers (61.2%) (see Table 1).

**TABLE 1:** Survey volunteer characteristics.

Variable	*N*	%
Gender (*n* = 331)		
Man/male	82	24.8
Woman/female	249	73.7
Age (*N* = 330)		
Under 25	12	3.6
25–35	18	5.5
36–45	23	7.0
46–55	27	8.2
56–65	70	21.2
66–75	133	40.3
76–84	43	13.0
85+	4	1.2
State or territory (*n* = 310)		
Western Australia	240	71.0
Victoria	68	20.1
New South Wales	2	0.6
Unknown	28	8.3
Locality (*n* = 310)		
Metro	238	70.4
Regional/remote	71	21.0
Combined (metro and regional)	1	0.3
Unknown	28	8.3
Volunteer type (*n* = 338)		
Mentor	131	38.8
Learning support volunteer	140	41.4
Both	67	19.8
Experience (*n* = 303)		
First time volunteer	96	28.4
More experienced	207	61.2
Unknown	35	10.4

#### 3.1.2 Impact of Volunteering

Overall, volunteers either ‘strongly agreed’ or ‘agreed’ that being part of EdConnect enhanced their mental health and wellbeing (*n* = 268, 89.4%) as well as their sense of community (*n* = 261, 87%). Two‐thirds of volunteers also reported that it had helped them develop new skills (*n* = 199), and over half felt volunteering enhanced their physical health (*n* = 175, 58.4%) (see Table 2). Open‐ended survey questions included comments about volunteer's mental health, for example:

‘I just enjoy meeting the children including those whom I don't interact with individually. I think it has been very good for my mental health’ (survey participant).

**TABLE 2:** Impact of volunteering.

	*n*	%
Enhances my mental health and wellbeing (*n* = 323)		
Strongly agree	155	48
Agree	134	41.5
Neither agree or disagree	31	9.6
Disagree	3	0.9
Enhances my physical health and wellbeing (*n* = 323)		
Strongly agree	77	23.8
Agree	115	35.6
Neither agree or disagree	118	36.5
Disagree	13	3.1
Helped me feel part of a community including the school (*n* = 323)		
Strongly agree	144	44.6
Agree	139	43.0
Neither agree or disagree	34	10.5
Disagree	6	1.9
Helped me develop new skills (*n* = 323)		
Strongly agree	82	25.4
Agree	132	40.9
Neither agree or disagree	93	28.8
Disagree	16	5.0

### 3.2 Interview Findings

Of the 30 potential interview participants who initially expressed interest, 22 were interviewed (73%); 13 (59%) were female. Two overarching themes were *structural impacts of motivations*; and *motivations and inter‐and intrapersonal impacts of volunteering*. Four subthemes help understand the *structural impacts of motivations*: *volunteer roles and responsibilities, volunteer qualities, school support* and *training opportunities*. The overarching theme *Motivations and inter‐and intrapersonal impacts of volunteering* included two subthemes: *giving back to society and helping students and staff* and *contributing to student social, emotional and academic outcomes*.

#### 3.2.1 Structural Impacts of Motivations

Participants volunteering experiences were impacted by their role and responsibilities. Their experiences reflected the level of training they received, the support received from the school and EdConnect and feedback they received about their volunteer role. Volunteers in this study were involved in a range of different roles with differing levels of responsibilities. Volunteer qualities also impacted experience and were related to motivators for volunteering. Volunteers also identified the challenge of their mentor role: ‘the students you're dealing with EdConnect in general are struggling and they're a challenge. So that's a challenge to try to help them (P4M)’.

##### 3.2.1.1 Volunteer Roles and Responsibilities

The length of time participants had been involved with EdConnect varied from one school term to 19 years, and there were three volunteer roles: Mentor (*n* = 14), Learning Support (*n* = 5) and a combination of both (*n* = 3). One volunteer explained their choice for the learning support role‐ ‘I'd just rather help everybody that needs some help, rather than do more time with just one’ (P13F). More volunteers chose to work within primary than secondary schools.

Following parental consent for student participation in the program, teachers nominated students and matched them to an EdConnect volunteer. Participants highlighted the importance of the tailored support, for example support was considered: ‘to have more benefit one‐on‐one, than what they're picking up in the classroom’ (P1F). Students were often taken out of class and received individual tuition that could involve games, art and craft, or extra assistance with subjects like English or mathematics.

Mentor roles included providing emotional support and friendship for those focussed on the social needs of the student. Mentors and Learning Support Volunteers also provided academic support to assist teachers with specific subjects. Contact hours with students varied from an hour and a half per week to two full days per week. Volunteers provided the opportunity for students to receive additional support and experiences in the classroom, for example, ‘we do things like clean out the chook pen, collect the eggs and stuff, those programs that are vital for kids, they wouldn't be doing that if you just had the one teacher to do it’ (P12F).

The professional and life experiences of volunteers varied widely and included several retired professionals including teachers, a medical practitioner, a psychologist, an engineer as well as some foster parents. While volunteers recognised the bounds of their professional expertise in this context, for example, ‘You're not a psychologist in there to solve any problems’ (P4M), those with extensive mental health backgrounds did discuss providing students with emotional support.

##### 3.2.1.2 Volunteer Qualities

All volunteers felt their life experiences were the most important skills they could share with the students and talked about how their personalities influenced their ability to establish rapport with students: ‘I've got a pretty outgoing personality; I can usually bring shy kids out of their little quiet space, and get kids involved’ (P12F). The need for empathy was mentioned several times and patience was listed as essential when working with the students in the mentoring and learning support roles for example: ‘the role is to listen and reflect, not to tell them what to do’ (P5M) and ‘Be patient with students and also be willing to listen and also like to share and you know, kind of heart to help support them not only the subject to the mentoring, but you know, everything, caring about the students’ (P18F).

Consistency was seen to be important when developing rapport and building a relationship with students. Being an adult that students could rely on was seen to be important: ‘I'm really just providing a regular adult who provides positive sense of wellbeing for her, and I play [sic] games with her, I read with her, and just encourage her to enjoy doing things’ (P3F). Similarly, patience and understanding from volunteers were attributed to a sense of wellbeing in students engaged with the EdConnect program: ‘I have the patience to give them the understanding they need. So, it gives them confidence, helps them to feel part of the classroom rather than being left behind’ (P21F).

Volunteers reflected on the importance of communication such as listening skills to enhance their relationship with their mentees and emphasised the need to ‘really listen to them, encourage them, build their self‐esteem’ (P8F). The ability to communicate with the students was considered essential to establish the connection and enrich the time spent: ‘We have good communication, and we have fun and I think she's gained somebody she knows is going to turn up every week just to spend time with her (P10F)’.

Promoting equality was relevant to one volunteer ‘I'm a friend and I treat him as an equal and he treats me as an equal’ (P15M). Sharing their life experiences, setting boundaries and limits was also identified as important, for example ‘I'm very firm with the children. I set boundaries, I have rules, it's strict, but it's done with love. And that makes them comfortable’ (P21F).

Male volunteers discussed their perception of the importance of male role models, especially in the primary school setting where there was often a higher proportion of female teachers and volunteers. Having a male to discuss issues was mentioned by several participants, for example:

‘I think that's the basis on which I see him is to sort of just be another male man to talk to about anything … and, and we have, we have the rule, but we talk about anything as I've said in my survey man to man, although he's nine and I'm 79’ (P15M).

##### 3.2.1.3 School Support

Volunteers described how their experience was related to the level of school support received. School support was discussed in relation to teacher and parent support. Volunteers valued feedback and recognition and discussed how some teachers identified the importance of the connection with students and suggested volunteers who were consistent and reliable were recognised as influencing a positive behaviour change in their students. Parents also gave feedback to the school identifying positive behaviour changes in their child since working with their mentor. Some volunteers explained that the school provided all the materials needed as well as a support person to contact if needed, for example: ‘the support I've had from the school and from EdConnect has been outstanding’ (P10F).

While overall support from the school and EdConnect was considered excellent, a few volunteers did discuss areas they thought support could be improved. A lack of a physical space was identified as an issue for a few volunteers who found it disconcerting working with students in the library, the canteen or outdoors in inclement weather.

A few volunteers were unclear about why they were mentoring a particular student and felt frustrated and would have appreciated more feedback from the school. As the following volunteer explained; ‘I thought he was just using it as an excuse to get out of class and I wasn't getting any feedback through the school as to what I should be doing or, you know, was this a valid role or not’ (P4M). Similarly, ‘it's just a show to me. That's my impression’ (P11M).

Volunteers were mostly very positive about the EdConnect program and were happy to encourage other potential volunteers to participate. They stressed the importance of commitment and consistency, of being neutral, non‐judgemental and keen to enjoy the school environment: ‘if someone wants to help these kids, I think it's very good that they do that, but need to go in with their eyes open’ (P11M).

Having fun and being available for one‐to‐two‐hours a week was discussed by several volunteers but taking the role seriously was also highlighted, for example: ‘they have to be prepared to work with kids who don't always do what you want them to do or ask them to do’ (P2F) and ‘don't go in there thinking you're gonna change a young person overnight. It's all about developing a relationship, be an active listener, provide advice without giving direction’ (P6M).

##### 3.2.1.4 Training (EdConnect Support)

Most volunteers were provided at least half a day of training which involved learning engagement strategies and understanding boundaries with the opportunity to participate in annual training. Incorporated in the training were scenarios that emphasised duty of care to the student and to self. Scenarios focused on abuse, depression, suicidal behaviours and eating disorders. Training included scenarios and discussions around unpromoted issues that volunteers may experience, as reflected by P7F: ‘you're there at the coalface’. Some volunteers were able to attend face‐to‐face training sessions once COVID‐19 restrictions eased. In addition, regular informal gatherings were organised by EdConnect for volunteers to meet and debrief with their peers and coordinators. Overall volunteers’ discussion around training was positive as highlighted by P17M:

‘The training, initially, the induction training was good. And then there's been ongoing training and a lot of it you get as feedback from the, from the coordinators and from the teachers and the children themselves. And the last couple of years were a bit more difficult with the COVID. But I still attended a couple of courses both online and face‐to‐face’.

#### 3.2.2 Motivations and Inter‐and Intrapersonal Impacts of Volunteering

Many volunteers expressed a combination of intrinsic and altruistic motivations which were also reflected in their perceptions of impacts of their volunteering experience. Two key sub‐themes help explain motivations for and impacts of volunteering: *giving back to society and supporting the school community* and *contributing to student social, emotional and academic outcomes*.

##### 3.2.2.1 Giving Back to Society and Supporting the School Community

Most volunteers who participated in the interviews were retired and felt they had the time to commit to supporting students and teachers as well as the skills to support and help students. Participants felt their life and professional skills offered them an opportunity to give to others and were proud of their contribution to helping students and supporting school staff. Volunteers talked about ‘being useful in society’ (P11M) and reflected they ‘still have a fair amount to give’ (P2F).

Participants discussed being a valued member of the school community. As well as helping others, some noted personal benefits as volunteering provided them with a sense of purpose and community, while others discussed how they were proud of their contribution: ‘I'm actually happy and proud of the role I did’ (P14M) and ‘You're helping someone, it makes yourself feel good, as well as whoever you're working with feel good about themselves. It's a win‐win situation’ (P1F).

A few volunteers discussed their concern for the current generation, and this motivated them to volunteer and support young people: ‘I think youth today are disorientated. They're disenchanted, don't seem to have direction’. Linked with that sentiment was the belief that ‘the children are the hope of our nation (P22M)’.

##### 3.2.2.2 Contributing to Student Social, Emotional, and Academic Outcomes

Motivations and impacts for volunteers also related to contributing to student social, emotional, and academic benefits, which contributed to feeling they were valued members of the school community. Participants discussed the satisfaction they felt helping students. Some participants noted student improvements academically, socially, emotionally and/or behaviourally and found this provided purpose and a sense of achievement from their volunteering role. For many, the interactions were also beneficial personally with some volunteers providing examples as to how the students also taught them new skills, for example: ‘they show you how to do things, and that gives them greater self‐esteem when they've taught you something’ (P1F). Similarly, another participant noted,

A very shy girl newly arrived from [East African Country], joined the Year 8 EAL [English as an additional language] class in July. At first, she could barely read a simple children's picture book, and she struggled to understand what the story was about. In just four months there has been a remarkable improvement. The memorable moment for me was a couple of weeks ago when she practised an oral presentation, and I saw just how much her confidence had grown. It was very special (survey participant).

Most participants described academic progress for their mentee which was associated with their volunteering. For some this was specific improvements such as an increase in grades while others described students increasing confidence and demonstrating general academic improvements. ‘One kid had three times his mark with my volunteering hours … we did the comparison between a one test he did it alone. And one test, the same test we did, he and me, I was guiding him. His result by himself. It was 9. His result with me. It was 28; Oh, wow’ (P9M). Another participant noted, ‘Yeah. And so that little bit of extra help. They think, Oh, that's not so bad, then. You know, and they, and they absolutely blossom, a lot of them’ (P7F).

Others discussed useful strategies such as playing games to enhance student learning:

‘They don't realise that they're learning numeracy and literacy by playing games’ (P1F).

Almost all participants described social and emotional benefits the volunteering afforded students. For some this related to enhancing student confidence and self‐esteem; being there to talk with the student; and improved behaviour, as highlighted:

‘Apparently she was a little bit difficult in the classroom. But apparently, when, I started s this support role, she softened an awful lot. And I found that she chatted the whole time. She was very enthusiastic. She wanted to do it. And it was like she was unloading a lot. That's what I felt anyway. You know, she just really wanted to chat, but we were doing the activity as well’ (P7F).

Volunteers described excitement and keenness from the students to engage especially when the relationship was consistent and transparent: ‘they [the students] get very excited when you turn up at the door because it's just somebody that cares about them’ (P1F). Some described a reduction in truancy, for example:

‘I'll never miss school on a Tuesday, because it's my time with you … attendance rates have improved, and I have worked with one girl for two whole years now. And the change in her was dramatic. I don't know if it's related to me, her circumstances or whatever, but she certainly has made huge gains both academically in attendance’ (P16F).

Social and emotional benefits for students were achieved by simply having someone to talk to, especially when their homelife presented challenges or when they struggled in the classroom:

‘And I did see that huge benefit for the kids that I was mentoring. It's just their home life was really difficult. And they had a lot of things seeing going on for them. And they would discuss some of those things that it's really hard in an hour’ (P8F).

‘And, yeah, so I think they gain a lot about well, and quite often the teachers will say, because sometimes we don't feel we've got made a difference. But the teachers will say, you know, that sometimes the child comes back in with a different mindset or a different attitude or, or they're so excited about you coming’ (P1F).

#### 3.2.3 Potential Improvements to the Program

Interview participants were generally happy with the EdConnect program, with the most common suggestion for improvement increased training for specific scenarios, for example ‘how do you react when a child says, Mum's too busy smoking weed on the couch’ (P3F). Similarly, volunteers felt being more informed about common problems like truancy, abuse and mental health issues would be helpful. Volunteers discussed their concern around the potential for disclosure of abuse with some suggesting they would benefit from a deeper understanding of mandatory reporting. Some volunteers felt they would benefit from more information about the student they were mentoring, for example: ‘If I knew more what their problem was, I'd base my activities around helping them with their problem’ (P19F).

## 4 Discussion

The findings of this study highlight the importance of school‐based volunteering for students, school staff and volunteers. Volunteers discussed improvements in student academic achievements, social and emotional health, and positive behaviour changes. The relationship between the volunteer and student was seen to be essential for the success of the volunteer experience. Youth mentoring programs have historically focussed on developing a relationship between the mentor and mentee through a range of activities [22]. Relationships between mentors and students have been associated with positive perceptions of the mentoring experience [16, 23] and academic outcomes [10]. Empathy, patience and listening without judgment have also been identified as qualities for volunteers [24]. Similarly, participants in the current study discussed the importance of their relationship with the student they mentored which was often built over time. Characteristics like listening, patience and understanding were highlighted as important to the relationship. A US study also found closeness, communication, engagement and compatibility to enhance mentor and student experiences [25].

Providing at‐risk students with a secure attachment experience which had potential to strengthen social and emotional adjustment, enhance emotional regulation and reduce stress was seen as an important part of the volunteer role in this study. Effective communication fostered trust and empathy between the mentor and mentee. Sound communication has been associated with stronger relationships between students, their parents and peers [23]. In the current study, several male volunteers explained the importance of role modelling and identity development, which is supported by Beyenhof [26] who found role modelling, personal appraisals and feedback, and promoting participation in activities increased student social and cultural capital. The current study supports evidence that youth may be more likely to attend school on the specific days they will spend time with their mentors [27].

Volunteers in this study were enthusiastic about the overall benefits of the program for students with most participants describing positive impacts including academic, social, emotional and behavioural gains of students they mentored. Despite this a few participants expressed that they felt some students took advantage of the program to ‘get out of class’. Nevertheless, volunteers in this study provided specific examples of academic improvement for children they mentored along with increased academic connections. Research from the US also demonstrated some positive impacts of school‐based mentoring programs on academic outcomes [10, 15]. Some volunteers also discussed the benefits of broader support for teachers, providing examples of helping in classrooms and working with disruptive students, enabling the teacher to concentrate on the whole class. Volunteers also discussed imparting specific skills such as mathematics, gardening, cooking and sport. In addition to potential benefits around individual support, these findings suggest that school‐based mentoring provides ongoing support for the school community by enhancing opportunities for all students through exposure to different activities.

In addition to benefits to students and the school community, volunteers themselves experienced a range of benefits, some of which may be associated with their motivations for being involved in the program. Volunteers in this study discussed intrinsic and altruistic motivations which were associated with the joy and satisfaction of being involved and of helping students and school staff. Many volunteers provided examples of how their volunteering experiences made them feel happy and connected. Ultimately motivations for, and benefits from, participating as a volunteer came from participants' experiences with the students, especially watching students develop academically, creatively, socially, and emotionally. Volunteers generally felt good about ‘giving back to society’ and helping others. This was supported by the quantitative data that found most volunteers felt part of the school community. This is consistent with the functional approach to volunteering especially the functions of values (to express pro‐social values) and social (to enhance social relationships) [1]. Studies of older people suggest they are more likely to volunteer for intrinsic and altruistic reasons [1, 2] while younger volunteers may also include extrinsic reasons including development of skills and experiences to further their career [1, 4]. Quantitative data from this study demonstrated most participants (89.5%) perceived volunteering with the EdConnect program contributed to positive wellbeing. Qualitative findings also supported a range of positive mental health and wellbeing associations with participation as a volunteer. A recent review found some positive associations between mental wellbeing and volunteering however cautions these findings dues to small study sizes and limited studies reported in the literature [28]. The findings of the current study are encouraging and suggest long‐term outcomes should be investigated.

Beyond the specific benefits this program had for students, school staff and volunteers, this program aligns with the concepts of a ‘school as a community hub’ [29]. A school‐based volunteer program such as EdConnect can support students to receive additional experiences and support not possible through formal education alone. Similarly, schools and volunteers benefit from differing partnerships. Schools with fewer students are not able to provide specialist teachers and so community volunteers with expertise may be able to fill these gaps [30]. Students and families who participate in a volunteer program may also be inspired to become future volunteers [31], thus promoting a cycle of social inclusion and ongoing academic and socioemotional benefits.

The findings of the current study reinforce the importance of the Australian National Standards for Volunteer Involvement to support safe, effective and inclusive volunteering [32]. Participants' spoke of the value of meaningful roles, that they are supported and developed and recognised. Findings provide insights into how volunteers and mentors perceive the value of their role, and the support afforded them by EdConnect and actors across the whole school community (i.e., teachers, parents, other mentors).

### 4.1 Strengths, Limitations and Future Research

While this study provides a good insight into volunteer perspectives on the EdConnect volunteer program, data from school staff and students would strengthen these findings. While the findings reflect perceptions around student improvements, matched data describing actual academic achievements are needed for definitive conclusions. Interview participants were mainly retired, older people which reflects the demographic of EdConnect volunteers. While survey data were collected from a broader age group, interview participants were mostly retired which may have biased the results. Age and current employment data were not collected from interview participants, this information was gained during the discussion. Females were more likely to complete the survey and participate in the interviews compared to males, however, this is also likely to reflect school‐based volunteer characteristics. The short time frame for data collection may have impacted recruitment of interview participants.

## 5 Conclusion

School‐based volunteer programs provide opportunity to enhance academic, social and emotional outcomes of students as well as providing support for school staff. In addition, the volunteering experience is associated with a range of social and emotional benefits to the volunteer. In this study, volunteering provided the opportunity to give back to the community, make a difference in a young person's life and increase life satisfaction for the volunteer. Future research is required with school staff and students to better understand the academic, social and emotional impacts volunteers have on individual students and the school community.

## Ethics Statement

Phase one and two of this project received ethics approval from Curtin University Human Research Ethics Committee: Phase 1 (formative research): HRE2021‐0121; Phase 2: HRE2021‐0437. Department of Education, Western Australia also provided approval to conduct research on Department of Education sites for phase 2 of the larger project (D22/0252219).

## Consent

All participants provided informed consent.

## Conflicts of Interest

The authors declare no conflicts of interest.

## Data Availability

The data that support the findings of this study are available on request from the corresponding author. The data are not publicly available due to privacy or ethical restrictions.
